# Expression of Matrix Metalloproteinase-1 in Alveolar Macrophages, Type II Pneumocytes, and Airways in Smokers: Relationship to Lung Function and Emphysema

**DOI:** 10.1007/s00408-014-9585-6

**Published:** 2014-05-03

**Authors:** Alison M. Wallace, Leanna B. Loy, Raja T. Abboud, Jeanine M. D’Armiento, Harvey O. Coxson, Nestor L. Muller, Steve Kalloger, Xing Li, W. Mark Elliott, John C. English, Richard J. Finley, Peter D. Paré

**Affiliations:** 1 St. Paul’s Hospital, University of British Columbia Center for Heart Lung Innovation, 166 - 1081 Burrard Street, Vancouver, BC V6Z 1Y6 Canada; 2 Division of Molecular and Pulmonary Medicine, Department of Medicine, College of Physicians and Surgeons, Columbia University, New York, NY USA; 3 Division of Respiratory Medicine, Department of Medicine, University of British Columbia, Vancouver, BC Canada; 4 Department of Radiology, Vancouver General Hospital, University of British Columbia, Vancouver, BC Canada; 5 Department of Pathology, Vancouver General Hospital, University of British Columbia, Vancouver, BC Canada; 6 Department of Surgery, Vancouver General Hospital, University of British Columbia, Vancouver, BC Canada

**Keywords:** Computed tomography, Emphysema, Expression, Immunohistochemistry, Metalloproteinase, Lung

## Abstract

**Background:**

An imbalance between proteolytic enzymes and their inhibitors is thought to be involved in the pathogenesis of chronic obstructive pulmonary disease. Matrix metalloproteinase-1, also known as interstitial collagenase, has been implicated as a potentially important proteinase in the genesis of chronic obstructive pulmonary disease and, more specifically, emphysema.

**Methods:**

We performed quantitative immunohistochemical assessment of matrix metalloproteinase-1 expression in the resected lung of 20 smokers/ex-smokers who had varying severity of airflow obstruction and emphysema and compared this with the lungs of 5 nonsmokers. Emphysema was measured using a morphometric measure of the lungs’ surface area/volume ratio and with qualitative and quantitative computed tomography (CT) measures of emphysema.

**Results:**

There were significantly more matrix metalloproteinase-1-expressing alveolar macrophages and type II pneumocytes as well as a greater percentage of small airways that stained positively for matrix metalloproteinase-1 in the lungs of smokers than in those of nonsmokers (*p* < 0.0001, *p* < 0.0001, and *p* = 0.0003, respectively). The extent of staining of type II pneumocytes and airways for matrix metalloproteinase-1 was significantly related to the extent of smoking (*p* = 0.012 and *p* = 0.013, respectively). In addition, the extent of matrix metalloproteinase-1 staining of alveolar macrophages was related to the lung surface area/volume ratio and to qualitative estimates of emphysema on CT.

**Conclusion:**

These findings suggest that cigarette smoking increases expression of matrix metalloproteinase-1 in alveolar macrophages as well as in alveolar and small airway epithelial cells. Smokers who develop emphysema have increased alveolar macrophage expression of matrix metalloproteinase-1.

**Electronic supplementary material:**

The online version of this article (doi:10.1007/s00408-014-9585-6) contains supplementary material, which is available to authorized users.

## Introduction

Cigarette smoke is the primary risk factor for chronic obstructive pulmonary disease (COPD), a disease state characterized by airflow limitation that is not fully reversible [1]. Not everyone who smokes develops COPD, and when it does occur, the pathological changes vary between patients and at different stages of the disease. The variable susceptibility and phenotypes suggest that there is a genetic component to the disease. Pathologic changes characteristic of COPD are found in the large airways, small airways (<2 mm), and alveoli. Chronic inflammation leads to fixed narrowing and obliteration of small airways and alveolar wall destruction; both changes result in airflow limitation.

More than 30 years ago, Hogg et al. [2] proposed that COPD is primarily a disease of the small airways based on the observation that the peripheral airways are the major site of increased resistance to airflow. Changes that contribute to the narrowing of the small airways include loss of alveolar and bronchiolar attachments, goblet-cell metaplasia, edema, inflammatory cellular infiltration, reduced surfactant, smooth muscle hyperplasia, and fibrosis [3]. The narrowing and obliteration of the small airways coupled with the loss of lung elastic recoil caused by parenchymal destruction leads to expiratory airflow limitation. The destruction of the lung parenchyma, which causes the lesions of emphysema, is believed to be due to an imbalance between the proteolytic and antiproteolytic processes in the lung. A number of proteolytic enzymes have been implicated in the disease process, including matrix metalloproteinases (MMPs), a family of zinc-dependent proteinases with the capacity to degrade both elastin and collagen.

MMP-1 is one of the most abundant proteases in the MMP family and is capable of degrading type I, II, and III collagens. Type I and III collagens are the most abundant proteins within the lung. The finding that MMP-1 transgenic mice overexpressing MMP-1 develop emphysematous changes in their lungs showed a direct contribution of MMPs to this proteolytic destruction [4, 5]. Further evidence from animal and human studies supports a role for collagenolytic enzymes and, in particular, MMP-1 in the pathogenesis of emphysema [6–9]. Genetic studies have also shown that MMP-1 polymorphisms are associated with COPD phenotypes [10, 11]. Alveolar macrophages, type II pneumocytes, and airway epithelial cells are known to express MMP-1 [7, 9]. In vitro studies have shown that cigarette smoke directly targets the MMP-1 promoter in human small-airway epithelial cells [12] and that this increase in expression is regulated via the extracellular regulated kinase/mitogen-activated protein kinase pathway [13]. Individual sensitivity to upregulation of expression of MMP-1 in response to cigarette smoke could contribute to individual susceptibility for COPD among smokers. Imai et al. [7] have previously identified alveolar macrophages and type II pneumocytes as two important cell types in the lung responsible for MMP-1 expression. Unfortunately, the study was limited by the small number of subjects (only two) who were long-term smokers but had normal lung tissue and therefore they were not able to compare the expression of MMP-1 in smokers with and without emphysema.

In the present study we quantified MMP-1 expression within the lung by immunohistochemistry and related MMP-1 expression to histological and computed tomography (CT) measures of emphysema in order to better evaluate the pathogenic role of this enzyme.

## Methods

Human lung specimens were obtained from 25 patients. Twenty lobes or lungs were obtained from smokers at the time of surgical resection for small (<3 cm diameter), stage I or II, peripheral tumors at the Vancouver General Hospital in Vancouver. Study participants who quit smoking 6 months or more prior to surgery were considered former smokers (*n* = 12); the remaining subjects in the smoking group were classified as current smokers (*n* = 8). The lungs or lobes of five never-smokers were obtained as controls from individuals who had surgical resection for various indications at St. Paul’s Hospital in Vancouver. Within the week prior to surgery the patients completed a questionnaire regarding smoking and respiratory symptoms and had measurements of subdivisions of lung volume, spirometry, and single-breath diffusing capacity as previously described and conforming to ATS standards [14]. Subjects provided informed consent. The study was approved by the University of British Columbia/Providence Health Care and Vancouver General Hospital Research Ethics Boards. Statistical analysis was performed using R 2.4.0 (Auckland, NZ). As a quantitative estimate of lifetime exposure to cigarette smoke, we used pack-years as ascertained in the preoperative interview. Unpaired *t*-tests were used to compare anthropometric, physiological, and morphological data of never-smokers with those of smokers and to make comparisons between former smokers and current smokers. Simple linear regression was used to estimate the associations between MMP-1 staining (alveolar macrophages, type II pneumocytes, and airways) and morphometric estimates of emphysema (Sa/V), as well as the qualitative and quantitative CT values. *P* values less than 0.05 were considered statistically significant. Details of the immunohistochemistry, image analysis, and CT analysis can be found in the Online Resource.

## Results

### Patient Characteristics

The demographics, smoking history, and pulmonary function of the subjects are presented in Table 1. In our sample, the never-smokers were significantly younger than the smokers (*p* = 0.027). Pulmonary function tests showed that the smokers had a reduced forced expiratory volume in 1 second (FEV_1_) compared with the never-smokers (*p* = 0.002) and a lower diffusing capacity for carbon monoxide (*p* = 0.033).

**Table 1:** Characteristics of study subjects

	Never-smokers (*n* = 5)	Smokers (*n* = 20)
Sex (male/female)	2/3	13/7
Age (years)	55.2 ± 12.4	68.7 ± 11.0
Smoking history (pack years)	0 ± 0.0	52.6 ± 33.3
FEV_1_ % predicted	109.0 ± 16.0	79.0 ± 16.5
DLCO % predicted	89.0 ± 4.5	75.0 ± 11.3

Data are mean ± SEM

### Cellular Localization of MMP-1

**Fig. 1:**
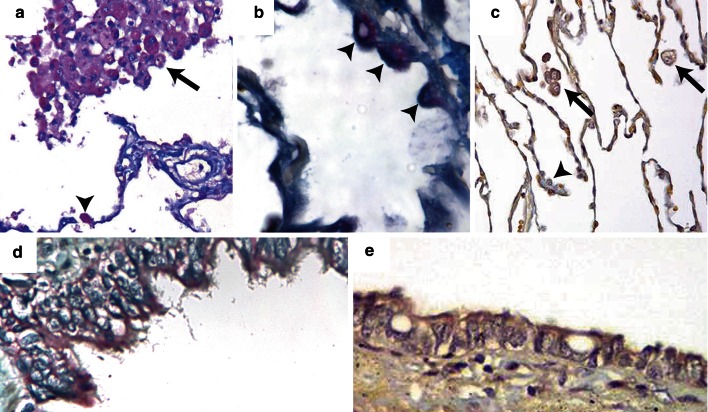
Immunostaining of the lungs of smokers with emphysema and never-smoker control subjects was performed with the use of a mouse monoclonal antibody specific to human MMP-1. Lung tissue from a smoker with emphysema showing positive MMP-1 staining in alveolar macrophages (**a**) and type II pneumocytes (**b**). Panel **c** shows a macrophage and a type II pneumocyte negative control. Panel **d** shows MMP-1-positive airway epithelial cells and panel **e** shows negative airway epithelium. *Arrows* indicate alveolar macrophage staining for MMP-1. *Arrowheads* indicate type II pneumocyte staining for MMP-1 (20 × for all pictures)

Immunohistochemistry was undertaken to identify the cell type(s) responsible for MMP-1 expression within the lung. MMP-1 was immunolocalized to alveolar macrophages (Fig. 1a), type II pneumocytes (Fig. 1a, b), and airways (Fig. 1d). Negative controls are shown in Fig. 1c, e.

### Comparison of MMP-1-Positive Cells in the Never-Smoker and Smoker Groups

Table 2 shows the amount of MMP-1-positive cells in the never-smoker and smoker groups. Smokers had significantly more MMP-1-positive stained alveolar macrophages (*p* < 0.0001), type II pneumocytes (*p* < 0.0001), and airways (*p* < 0.0003). Type II pneumocytes did not stain positively for MMP-1 in the lungs of never-smokers, staining was weakly positive in alveolar macrophages, and only one nonsmoker had any positive airway staining.

**Table 2:** MMP-1 positivity in the never-smoker and smoker groups

	Never-smokers (*n* = 5)	Smokers (*n* = 20)	*p* value
Alveolar macrophages	1.38^a^ ± 0.60	9.68^a^ ± 1.45	<0.0001
Type II pneumocytes	0.0 ± 0.0	0.78^a^ ± 0.17	<0.0001
Airways^b^	10 ± 10	77 ± 6	0.0003

Data are mean ± SEM. The values for alveolar macrophages and type II pneumocytes represent the volume fractions of positively staining cells over total lung volume

^a^The volume fractions have been multiplied by 10^6^ for convenience

^b^MMP-1 positivity of airways is expressed as the percentage of the airways positive for MMP-1

To determine if the amount of MMP-1 staining and qualitative CT score were affected by current smoking status, we divided the smoking group into former smokers and current smokers. Table 3 shows that there were no significant differences between the two subgroups in terms of the amount of MMP-1 staining in any of the cell types, suggesting that smoking cessation does not result in a decrease in MMP-1 expression. In addition, the quantitative CT score did not differ significantly between the former smokers and current smokers (Table 3).

**Table 3:** Staining, expressed as volume fraction and percent positive airways, and quantitative CT scores in the smoking group according to smoking status

	Former smokers^a^ (*n* = 12)	Current smokers (*n* = 8)	*p* value
Alveolar macrophages	10.68^b^ ± 0.195	8.19^b^ ± 0.218	0.203
Type II pneumocytes	0.86^b^ ± 0.25	6.62^b^ ± 0.23	0.293
Airways	84 ± 4	67 ± 12	0.073
Overall emphysema %^c^	25.1 (14.3–31.7)	45.4 (32.8–49.7)	0.234
Severe emphysema %^d^	2.3 (0.4–13.4)	8.1 (4.9–21.3)	0.181

Data are mean ± SEM or (interquartile range)

^a^Study participants who quit smoking 6 months or more prior to surgery

^b^The volume fractions have been multiplied by 10^6^ for convenience

^c^Defined using a density mask cutoff of −855 Hounsfield units

^d^Defined using a density mask cutoff of −910 Hounsfield units

### Amount of Staining and Emphysema

We analyzed whether staining in each of the cell types was related to the surface area/volume ratio (a low ratio indicates emphysema), as well as to the qualitative and quantitative CT grades of emphysema. Since smoking had such a profound effect on MMP-1 expression, we limited the analysis to the 20 subjects with a smoking history. This addresses the question of whether smokers who develop emphysema have increased expression of MMP-1 relative to smokers who do not. The surface area/volume ratio was significantly negatively related to alveolar macrophage staining (*r* = −0.43, *p* = 0.029) but not to type II pneumocyte staining (*r* = −0.29, *p* = 0.11) or airway staining (*r* = −0.25, *p* = 0.14).

The distribution of qualitative CT emphysema scores was grade 0 (*n* = 1), grade 1 (*n* = 9), grade 2 (*n* = 6), and grade 4 (*n* = 1). For ease of comparison, the one subject with grade 0 and the one subject with grade 4 were excluded from the analysis; there were no subjects in the grade 3 group. The amount of MMP-1 staining in alveolar macrophages in subjects with grade 2 emphysema was significantly greater than that of those with grade 1 emphysema (*p* = 0.03). There was no significant difference between the amounts of staining in type II pneumocytes and airways as a function of the qualitative grade of emphysema. There were also no significant associations between quantitative CT values and the amount of positively stained cells. When adjusted for smoking status, the overall emphysema score (defined using the density mask cutoff of −855 Hounsfield units) showed a borderline association with staining of type II pneumocytes (*p* = 0.073), with higher emphysema being positively related to staining for MMP-1.

## Discussion

The results of this study provide some support for the claim by D’Armiento et al. [7] that MMP-1, a nonelastolytic collagenase, may play a pathogenic role in emphysema. In addition, the results are concordant with our previous finding of a significant relationship between MMP-1 mRNA expression by alveolar macrophages and the extent of emphysema measured by CT scan [9]. In the present study we demonstrated MMP-1 immunolocalization to alveolar macrophages, type II pneumocytes, and airways. Furthermore, MMP-1 staining was significantly increased in smokers; in the control group there was no staining of type II pneumocytes and only one of five individuals showed any staining of airway epithelium. Interestingly, the amount of MMP-1 staining did not differ between the current and former smokers, suggesting that cigarette smoke induces long-term changes in the expression of this proteinase that persist after smoking cessation. Although MMP-1 staining was increased in all smokers, there was significantly more staining of alveolar macrophages in individuals with a higher emphysema score. This is a novel observation. Previously, when MMP-1 expression in the lungs of smokers with emphysema was compared to that of nonsmokers, substantial differences were found in MMP-1 expression by alveolar macrophages and type II pneumocytes [7]. However, only two smokers without emphysema were included in the study and thus the contribution of smoking versus emphysema could not be distinguished.

The inflammatory mediators and proteases that initiate and sustain proteolytic injury to the lung remain unclear. Multiple studies have shown that the numbers of inflammatory cells in the lung are markedly increased in response to cigarette smoke exposure [15]. Our findings show that the macrophages in smokers’ lungs express increased MMP-1 and, furthermore, that the number of positive macrophages increases with the severity of emphysema. However, it is not known whether this initial inflammatory reaction is harmful or to what extent resultant upregulation of other proteolytic enzymes also causes lung destruction. For example, the expression of MMP-12, an elastolytic enzyme, has also been shown to be increased in individuals with emphysema [9]. Although transgenic upregulation of MMP-1 in mice leads to emphysema [4], knockout of the MMP-12 gene prevents the development of emphysema in mice exposed to cigarette smoke [16], implicating both of these proteases in the process. In this study we focused on MMP-1 because of its ability to degrade type III collagen, the major structural component of the alveolar ducts and septa, which are key regions subjected to proteolytic destruction in emphysema [8].

Our results are also supported by the study of Gosselink et al. [17]. Those investigators used laser capture microdissection to obtain mRNA from the alveolar region of the peripheral lung as well as from the small airways. They showed that MMP-1 expression was increased in the lung parenchymal tissue as a function of increasing GOLD COPD severity. There was no differential expression of MMP-1 in the airways as a function of airflow obstruction.

This study has limitations. It is an observational study examining the expression of MMP-1 within various cell types within the lung in a small sample of subjects. We measured the volume fraction of the lung parenchyma that contained positive staining alveolar macrophages and type II pneumocytes rather than the fraction of these cell types that were positive. Therefore, it is possible that an increase in the total number of alveolar macrophages and type II pneumocytes in the lungs of the smokers and in the more emphysematous subjects could contribute to the differences. Although it is true that there are more alveolar macrophages and type II pneumocytes in the lungs of smokers, we do not believe that this is the main source of the difference. The alveolar macrophages and type II pneumocytes of nonsmokers displayed little or no staining; the signal was not due simply to increased cell number. Similarly, there were more MMP-1-positive alveolar macrophages and type II pneumocytes in subjects with more severe emphysema, not simply more cells.

As discussed above, a potential weakness of this study is that we examined the expression of only one enzyme thought to play a destructive role in the disease process. Furthermore, due to our limited sample size, we were not able to fully examine the relationship between MMP-1 expression and all of the GOLD stages of disease or grades of emphysema. Our never-smoker controls were younger than our smokers, with and without emphysema, primarily due to the difficulty of obtaining normal samples from patients in their fifth and sixth decade of life, when emphysema normally affects patients.

In summary, our findings further support the proposal that inflammatory cells (alveolar macrophages), as well as type II pneumocytes and airway epithelium, are a significant source of MMP-1 and that this enzyme plays a role in the pathogenesis of emphysema, even after smoking cessation. In addition, increased MMP-1 expression correlates with more severe disease, suggesting that MMP-1 plays a role in the disease process and that regulation of this enzyme may prove to be a valuable therapeutic intervention.

## Electronic supplementary material

Below is the link to the electronic supplementary material.

Supplementary material 1 (DOC 43 kb)
