# Supplementary material for: Expression of Matrix Metalloproteinase-1 in Alveolar Macrophages, Type II Pneumocytes, and Airways in Smokers: Relationship to Lung Function and Emphysema
Source: Lung. 2014 May 3;192(4):467–72. doi: 10.1007/s00408-014-9585-6 (PMC4104162; doi:10.1007/s00408-014-9585-6)
Supplement: Supplementary file 1 — Supplementary material 1 (DOC 43 kb) [file 408_2014_9585_MOESM1_ESM.doc]

**ONLINE RESOURCE**

**METHODS**

***Immunohistochemistry***

Following surgery, the resected specimens were transferred directly from the operating room to the laboratory. The specimens were inflated with Bouin’s fixative or formalin at a constant distending pressure of ~25 cm of water and immersed in formalin overnight. Post fixation, each specimen was cut into slices with 5-15 mm thickness in the axial plane. A grid of 2 × 2 cm squares was superimposed over each lung slice; squares were randomly selected and the tissue beneath it was excised and embedded in paraffin. Four tissue samples were randomly selected from each of the subjects and were sectioned at 4 μm thickness onto coated glass slides and stained for MMP-1 using the alkaline phosphatase anti-alkaline phosphatase (APAAP) method. Briefly, slides were deparaffinized in Citrisolv, rehydrated in a graded alcohol series, and rinsed with water. Antigen retrieval was performed using 10 mM Tris-HCl solution at pH 7.5 in an autoclave, after which the slides and buffer were cooled to room temperature for 30 minutes and subsequently rinsed in Tris-buffered saline (TBS, pH 7.6). Universal Protein Block Serum-Free (DAKOCytomation, Mississauga, ON) was used to prevent non-specific binding of the antibody during an incubation at room temperature for 20 minutes. An anti-MMP-1 antibody (IM35L, Oncogene/ Calbiochem, La Jolla, CA) at 5 μg/mL concentration in 1% bovine serum albumen (BSA, Sigma, St. Louis, MO) and TBS was then added to the tissue sections and they were placed in a humidified chamber overnight. As a negative control, sections were processed with an isotype matched nonspecific mouse IgG_2a_ (BD Biosciences Pharmingen, Mississauga, ON) in the absence of the primary antibody. Slides were rinsed for 20 minutes in TBS on an orbital shaker at a speed of 100 RPM to reduce non-specific binding of primary antibody. A secondary antibody consisting of a Rabbit anti-mouse IgG (DAKOCytomation) was added to the slides and incubated for 30 minutes followed by a 10 minute rinse in TBS. The APAAP (DAKOCytomation) enzyme system was subsequently added to the slides and incubated for a further 30 minutes followed by a 10 minute rinse in TBS. This was followed by using Naphthol/New Fuschin as the chromogenic substrate (DAKO Handbook) for 20 minutes. All immunohistochemical preparations were counterstained with hematoxylin (Sigma), dehydrated and a cover slip was applied.

***Image analysis***

Images were captured using a Nikon Eclipse E600 microscope fitted with a SPOT camera. The image analysis was performed using Image Pro Plus 4.0 (Media Cybernetics, Silver Spring, MD). Histologically, the severity of emphysema was determined by analyzing 6 random fields/slide at 24x magnification and by using a locally developed software program. Briefly, each image was binarized and a grid of lines was superimposed on the image. The program automatically counts the number of intersections between the superimposed lines and the alveolar walls (i.e. tissue–air interface, Σ1), the number of total line endpoints that fall on tissue (i.e. ΣP tissue), as well as the total number of line endpoints (i.e. ΣP total). Estimates of the percentage of tissue in the lung (volume fraction) and the surface area to volume ratio of the alveolar walls were calculated using the following equations:

(1) Volume Fraction of Tissue (Vv tis) = ΣP tissue / ΣP total

(2) Surface density (Sv)= (4 x Σ1) / (Grid Length x ΣP tissue)

(3) Surface Area / Volume Ratio (SA/Vol) = Sv x Vv tis

Surface area/Volume ratio for each of the samples was corrected for shrinkage. The shrinkage factor was determined by measuring the length of one side of the blocks prior to fixation processing and then dividing by the length of one side of the cut sections after processing.

The histology was examined by observers who were blinded to the origin of the material. The number of MMP-1 positive airways and cells were determined on images taken at 120x magnification. From each slide, 30 random fields were sampled. Positive airways were determined by any positive staining in any airway compartment (epithelium, lamina propria, or adventitia) and expressed as percent positive airways/slide. The degree of MMP-1 staining in alveolar macrophages and Type II pneumocytes was determined by applying a grid of points over the images and manually counting the points that fell on positively stained cells. The volume fraction of positively stained cells (Vv alveolar macrophages and Vv Type II pneumocytes) was calculated using equation 1 where ΣP cells (points on positively stained cells) is substituted for ΣP tissue and ΣP total is the total number of points on the alveolar walls and airspaces.

***Computed tomographic (CT) analysis***

The scans were evaluated for the extent and severity of emphysema using both a qualitative and a quantitative method. For the qualitative assessment, two independent observers graded the extent of emphysema using a 6-point scale (0: no emphysema and 5:>75% emphysema). CT scans for seventeen subjects (9 former smokers and 8 current smokers) were available for qualitative analysis. The quantitative analysis of the lung parenchyma was performed using custom software (EmphylxJ, Vancouver, BC, Canada) as previously described [1]. Briefly, the lung parenchyma was segmented from the chest wall and large central blood vessels using a modified border tracing algorithm with a prior position-knowledge algorithm. Lung volume was calculated by summing the segmented pixel area in each slice and multiplying by the slice thickness to acquire the total lung volume. The CT density of the lung (g/ml) was estimated from x-ray attenuation of each of the CT voxels [2]. Using this analysis, we obtained two quantitative computerized measures of emphysema employing the density mask cut-offs of −855 Hounsfield units (FracVol 6; overall emphysema) and -910 Hounsfield units (FracVol 10.2; severe emphysema), which are appropriate for this CT acquisition technique [3]. Digital CT scans for thirteen subjects (7 former smokers and 6 current smokers) were available for quantitative analysis. The reported values are the percent of voxels that fell below the cut-offs.

**REFERENCES**

1. Coxson HO, Rogers RM, Whittall KP, D’Yachkova Y, Pare PD, Sciurba FC, Hogg JC (1999) A quantification of the lung surface area in emphysema using computed tomography. *Am J Respir Crit Care Med* 159:851–856
2. Coxson HO, Mayo JR, Behzad H, Moore BJ, Verburgt LM, Staples CA, Pare PD, Hogg JC (1995) Measurement of lung expansion with computed tomography and comparison with quantitative histology. *J Appl Physiol* 79:1525–1530
3. Yuan R, Nagao T, Paré PD, Hogg JC, Sin DD, Elliott MW, Loy L, Xing L, Kalloger SE, English JC, Mayo JR, Coxson HO (2010) Quantification of lung surface area using computed tomography. *Respir Res* 11:153-161
